# The association between parenting stress and young children’s social–emotional competence: a moderated mediation model

**DOI:** 10.3389/fpsyg.2026.1644623

**Published:** 2026-02-16

**Authors:** Huijuan Di, Cuicui Hao, Yuan Liu, Jiaxuan Jiao, Deqiang Wang, Huanhuan Li

**Affiliations:** 1 College of Teacher Education, Hebei Normal University, Shijiazhuang, China; 2 College of Home Economics, Hebei Normal University, Shijiazhuang, China; 3 College of Educational Science, Xinjiang Normal University, Urumqi, China

**Keywords:** China, family socioeconomic status, mindful parenting, parenting stress, young children’s social–emotional competence

## Abstract

**Background:**

As a core construct of early psychological development, the quality of young children’s social–emotional competence is not only directly associated with relationship building and emotion regulation, but also shapes lifelong adaptability through cognitive control mechanisms. However, in the face of the significant increase in young children’s social–emotional problems globally, although existing studies have confirmed the negative correlation between parental stress and young children’s social–emotional competence, systematic studies on the potential association pathways in young children aged 3–6 are still lacking.

**Methods:**

A cross-sectional study design was used; 402 parents of young children aged 3–6 years in Hebei province were asked to complete online questionnaires, including the Parenting Stress Questionnaire, the Young Children’s Social–emotional Competence Questionnaire, and the Mindful Parenting Questionnaire. The mediation model with moderation was tested using the PROCESS plug-in for SPSS.

**Results:**

(1) Parenting stress is significantly and negatively associated with young children’s social–emotional competence. (2) Mindful parenting plays a partial mediating role in the relationship between parenting stress and young children’s social–emotional competence (indirect effect = −0.02, 95% CI [−0.033, −0.001]). (3) Family socioeconomic status played a significant but weak moderating role in the association between parenting stress and young children’s social–emotional competence, and this negative moderating role was stronger in families with high family socioeconomic status (*β* = −0.03, *p* < 0.005, 95% CI = [−0.050, −0.002]).

## 1 Introduction

Social–emotional competence refers to children’s mastery and application of a set of core non-cognitive competencies related to self-adaptation and social development (Xiong et al., 2023), and plays a crucial role in both the development of the individual and the healthy functioning of society (Chernyshenko et al., 2018). It has been found that preschoolers’ social–emotional competence is significantly associated with their future academic, social, and psychological development. Specifically, young children with higher social–emotional competence tended to have better relationships with parents, friends, and teachers and achieved more academic and social success. Conversely, young children who lack social–emotional competence are often at risk for reduced social opportunities, rejection, withdrawal, behavioral disorders, and achievement problems (McCabe and Altamura, 2011). However, with the rapid development of Chinese society and the transformation of educational concepts, Chinese parents’ expectations for child-rearing have been continuously rising, leading to high-competition educational phenomena such as “chicken parenting” and “involution,” which in turn have increased the pressure on parents. Therefore, given the complexity, high demands, and unpredictability of current society, children need certain cognitive, social, and emotional skills to adapt to their social environment (Miyamoto et al., 2015). Based on this, the development of children’s social and emotional ability needs to be paid more attention, and it is necessary to explore the factors influencing it, especially parenting factors.

The family stress model posits that adverse circumstances like financial strain do not directly impact children’s development. Instead, they amplify parental stress and psychological distress, which undermines positive parenting behaviors and ultimately harms children (Conger and Donnellan, 2007). Thus, this study focuses on the model’s central dependent variable: parenting stress. Parenting stress refers to a range of unpleasant physical and psychological reactions that arise in parents as they adjust to the demands of the parenting role (Deater-Deckard, 2008). Generally, parents experience varying degrees of parenting stress throughout child-rearing stages, with preschool-aged children showing particularly pronounced stress perception (Anthony et al., 2005). Theoretically, parents experiencing high parenting stress may develop diminished capacity for positive parenting behaviors, impairing their emotional and behavioral support functions for children. This manifests as alienation, avoidance responses, and varying degrees of child abuse, indirectly hindering the development of children’s socio-emotional abilities (Ponnet et al., 2013). Furthermore, according to the Parent Effect Model of Child Development, parents’ inherent traits and psychological states—particularly their stress experience—directly exert profound impacts on children’s psychological and behavioral development (Stone et al., 2016). Extensive research confirms that parenting stress significantly predicts problematic behaviors in children, including internalizing issues (depression, anxiety, social sensitivity disorders) and externalizing behaviors (aggression, defiance) (Tan et al., 2012), while also impairing socio-emotional development. Although a lot of researches have proved that there is a significant negative correlation between parenting stress and children’s social–emotional competence, most of them still focus on the direct correlation. According to the family stress model, the core of the research should be the mediating mechanism of “parental behavior” which connects parental stress with children’s development.

In recent years, research has increasingly focused on empowering parents to implement more effective parenting strategies. Within this trend, mindful parenting—a novel approach integrating mindfulness principles into daily parenting practices—has emerged as a promising and highly practical research field. Mindful parenting refers to parents ‘ability to purposefully and non-judgmentally observe their own and their children’s internal states during parent–child interactions, enabling conscious responses (Kabat-Zinn, 1994). Theoretical frameworks therefore identify mindful parenting as a key mediator in this process. This is because chronic parenting stress, acting as a psychological resource drain, can trap parents in a “psychological rigidity” state, making it difficult to maintain emotional stability and focused attention, thereby systematically eroding their capacity to practice mindful parenting (Gouveia et al., 2016). Stressful parenting often leads to automatic, reactive, and impatient behaviors—precisely counterproductive to the awareness, acceptance, and conscious responsiveness advocated by mindful parenting. Conversely, when parents practice mindful parenting, they create the most fertile ground for developing children’s social–emotional competencies (Wang et al., 2023). By fostering sensitive responses, positive parent–child communication, and modeling positive emotions, mindful parenting directly supports the development of children’s emotional regulation, empathy, and social skills (Duncan et al., 2009). Therefore, we logically deduce that the negative effects of parenting stress on children’s social–emotional competence are not direct, but indirect, through weakening parents’ mindfulness parenting level.

Furthermore, according to the stress process theory (Pearlin, 1989), a family’s socioeconomic status may serve as a crucial moderating factor in buffering or exacerbating the negative consequences of stress. The resources a family possesses (such as socioeconomic status) can either cushion or amplify stress transmission (Conger et al., 2010). Family socioeconomic status (SES) refers to the hierarchical level of value resources a family holds within its social context, reflecting differences in resource allocation among families and profoundly influencing individual development (Matthews and Gallo, 2011). Generally, parents from different family economic backgrounds exhibit variations in social resources and stress coping strategies when facing parenting stress, resulting in distinct negative impacts on young children. Specifically, parents from high socioeconomic status families typically possess abundant economic resources and sufficient social support to cope with stress, enabling them to adopt more proactive coping strategies that effectively alleviate pressure. Conversely, parents from low socioeconomic status families, constrained by financial poverty and limited social support, may adopt more passive coping mechanisms. This could trigger a snowball effect, causing stress to accumulate and spread like a snowball, thereby potentially leading to more detrimental outcomes for the development of children’s social–emotional abilities. Meanwhile, Cassells and Evans (2017) demonstrated that family socioeconomic status profoundly influences parenting stress through three key mediating factors: depressive mood, family structure, and neighborhood environment. Notably, a significant negative correlation exists between parenting stress and socioeconomic status (Raikes and Thompson, 2005). Furthermore, children from lower socioeconomic backgrounds exhibit more frequent symptoms of mental disorders and social adaptation difficulties compared to their affluent counterparts (Bradley and Corwyn, 2002). This finding aligns with Kuo et al.’s (2020) research, which revealed a positive correlation between family socioeconomic status and children’s socioemotional competence. Therefore, this study adopts a moderated mediation model, examining the mediating role of mindful parenting and the moderating effect of family socioeconomic status in this relationship between parenting stress and children’s social–emotional competence.

**Figure 1:**
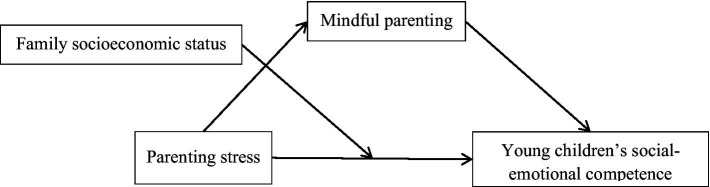
Research theoretical model.

In summary, the present study proposes a moderated mediation model (see Figure 1) based on existing theories and related research, aiming to explore the mediating role of mindful parenting in the association between parenting stress and young children’s social–emotional competence and to examine the moderating role of family socioeconomic status in the model.

## 2 Materials and methods

### 2.1 Participants

This study adopted a cross-sectional design and recruited 416 parents of children from multiple kindergartens in Hebei Province, China as participants. After eliminating questionnaires with logic problems and incomplete information, 402 valid data were finally obtained (see Table 1 for specific information). All questionnaires were completed by the children’s primary caregivers, including 78.9% mothers, 21.1% fathers, to ensure the accuracy and consistency of the reports. The age of the subject young children ranged from 3 to 6 years with a mean age of (4.5 ± 0.95) years, of which 199 (49.5%) were boys and 203 (50.5%) were girls. Hebei Province, surrounding the capital Beijing of China, is a core component of the Beijing-Tianjin-Hebei urban agglomeration, with its demographic structure, economic development level, and socio-cultural characteristics being highly representative of northern China. The province encompasses both large industrial cities such as Shijiazhuang and Tangshan, as well as extensive urban and rural areas, forming a microcosm of China’s social transition period. Therefore, selecting Hebei Province as the sample source helps us delve deeper into the impact of family factors on child development under the background of rapid urbanization and modernization in China. The research findings hold significant reference value for understanding population groups in northern China and even regions at similar developmental stages.

**Table 1:** Summary of basic information of samples (*n* = 402).

Items	Demographic statistical variables	Frequency (*n*)	Percentage (%)
Child gender	Male	199	49.5
	Female	203	50.5
Child age	3–4 years	89	22.1
	4–5 years	75	18.7
	5–6 years	184	45.8
	6–7 years	54	13.4
Number of siblings	1	174	43.3
	2	205	51.0
	3 or more	23	5.7
Parent–child relationship	Father	85	21.1
	Mother	317	78.9
Father’s education level	Junior high school or below	3	3.5
	Technical secondary school/high school	11	12.9
	College	19	22.4
	Bachelor’s degree	39	45.9
	Master’s degree or above	13	15.3
Mother’s education level	Junior high school or below	20	6.3
	Technical secondary school/high school	25	7.9
	College	68	21.5
	Bachelor’s degree	174	54.9
	Master’s degree or above	30	9.5
Family monthly income	≤3,000 RMB	30	7.5
	3,000–5,000 RMB	78	19.4
	5,000–8,000 RMB	88	21.9
	8,000–10,000 RMB	81	20.1
	≥10,000 RMB	125	31.1

### 2.2 Measures

#### 2.2.1 Family socioeconomic status

Social economic status (SES) is a measure of the social and economic status of family members, which divides individuals into social tiers based on the amount of social resources they can access or control, concerning social resources that generally include factors such as family members’ educational attainment, income level, and occupational prestige (Ren, 2010). In this study, we refer to the research results of Liang et al., 2021 to determine the measurement indicators of family socioeconomic status, including both parents’ monthly family income level and parents’ education level. The standardized monthly family income and parents’ education level were subjected to principal components analysis to calculate factor loadings and the first characteristic root (Xu et al., 2024). The formula is, family socioeconomic status = (β1*Z primary caregiver’s education + β2*Z family income)/the characteristic root of the first factor, where β1 and β2 are the factor loadings of each indicator. A higher score for family socioeconomic status represents a higher socioeconomic status of the family.

#### 2.2.2 Parenting stress

The Parenting Stress Index-Short Form (PSI-SF) measures parenting stress using a shortened version of the Parenting Stress Scale developed by Abidin (1995). The scale has 36 entries and includes three dimensions: parental distress, dysfunctional parent–child interaction, and difficult child. Each entry is scored using a five-point scale from 1 to 5. The Parenting Stress Score is equal to the total score of the three dimensions of parental distress, dysfunctional parent–child interaction, and difficult child, with higher scores representing greater parenting stress. Parents with total scores equal to and above the percentile rank of 90 and below the percentile rank of 15 were considered to be in the high and low parenting stress groups, respectively, based on the questionnaire scoring guide of the Parenting Stress Index-Short Form (PSI-SF). In this study, the continuous total score was retained for all inferential analyses to maximize statistical power and model sensitivity. The internal consistency alpha coefficient of the total scale in this study is 0.96, with 0.90 for parental distress, 0.95 for dysfunctional parent–child interaction, and 0.89 for difficult child.

#### 2.2.3 Mindful parenting scale

Using the Mindfulness Parenting Scale developed by Huang (2024). The scale consists of 9 entries and each entry is scored on a 5-point scale, strongly agree = 1, agree = 2, unsure = 3, disagree = 4, strongly disagree = 5. Higher scores on the scale indicate higher levels of positive parenting. The overall Cronbachαcoefficient for the scale in this study was 0.72, indicating high internal consistency of the scale.

#### 2.2.4 Young children’s social–emotional competence

Children’s social–emotional competence was measured using the Chinese Inventory of Children’s Social–emotional Competence (CICSEC) developed by Li et al. (2020). The scale consists of four dimensions: cognitive control, emotion expressivity, empathy and prosocial behaviors, and emotion regulation, with 30 entries (of which 13–24 are reversed), and each entry is scored on a 5-point scale. To align with the norm-referenced standard used in this study (Wang et al., 2019) and given the non-normal distribution characteristic of psychometric data, we followed the standardization procedure of the scale. Using the non-parametric percentile method, we transformed the raw scores of social–emotional competence into total scores and standardized scores. Higher total, standardized social–emotional competence scores represent better development of young children’s social–emotional competence. If the total score of young children’s social–emotional ability is 1 standard deviation lower than the average level of healthy children, it indicates that the child’s social–emotional ability has a slight risk of lag; If a child’s total social–emotional score is 2 standard deviations below the average of healthy children, it indicates that the child is at risk for a significant lag in social–emotional competence (Wang et al., 2019). The overall Cronbach *α* coefficient for this scale in this study was 0.88, with alpha coefficients of 0.89, 0.788, 0.92, and 0.80 for cognitive control, emotion expressivity, empathy and prosocial behavior, and emotion regulation, respectively.

### 2.3 Statistical analysis

Descriptive statistics, differential analysis, and regression analyses were performed in this study using SPSS 27.0, and mediation tests and mediation with moderation tests were performed using the SPSS macro program PROCESS prepared by Hayes.

## 3 Results

### 3.1 Common method bias test

Data were tested for common method bias using Harman’s single-factor test (Zhou and Long, 2004). The results show that there are 18 factors with eigenvalues greater than 1. The first factor explains 23.48% of the variance, which is less than the critical value of 40%, therefore, the data in this study do not suffer from serious common bias.

### 3.2 Descriptive statistics and correlation analysis

Table 1 presents the results of the mean, standard deviation, and correlation analysis for each variable. Pearson correlation analysis of the variables revealed (see Table 2): there was a significant negative correlation between parenting stress and young children’s social–emotional competence, positive mindful parenting, and family socioeconomic status; young children’s social–emotional competence was positively correlated with mindful parenting and family socioeconomic status.

**Table 2:** Results of descriptive statistics and correlation analysis for each variable.

Item	*M*	*SD*	1	2	3	4	5	6
1 Gender	-	-	1					
2 Age	4.51	0.98	-	1				
3 Parenting stress	2.25	0.60	−0.06	0.10*	1			
4 Young children’s social–emotional competence	3.48	0.44	0.02	−0.08	−0.66**	1		
5 Mindful parenting	2.80	0.58	0.03	0.00	−0.22**	0.24**	1	
6 Family socioeconomic status	8.47	2.30	−0.02	0.04	−0.24**	0.18**	0.02	1

*p < 0.05, **p < 0.01.

### 3.3 Testing for moderated mediation models

Based on the clarification of the degree of correlation of each variable, this study used SPSS and PROCESS 4.1 programs to analyze the pathways of each variable affecting the young children’s social–emotional competence.

#### 3.3.1 Mediation model test for mindful parenting

First, the mediating effect of mindful parenting was tested using Model 4 in the Hayes procedure. The results showed (see Table 3) that after controlling for child gender and age, parenting stress negatively predicted positive mindful parenting ( *β* = −0.21, *p* < 0.001) and positive mindful parenting positively predicted young children’s social–emotional strength ( *β* = 0.08, *p* < 0.001).

**Table 3:** Mediation model test.

Predictor variable	Equation 1		Equation 2		Equation 3	
	Young children’s social–emotional competence		Mindful parenting		Young children’s social–emotional competence	
	*β*	*t*	*β*	*t*	*β*	*t*
Parenting stress	−0.48	−16.99***	−0.21	−4.50***	−0.47	−16.30***
Mindful parenting					0.08	2.67***
Gender	−0.01	−0.41	0.02	0.28	−0.01	−0.45
Age	−0.01	−0.51	0.02	0.56	−0.01	−0.59
*R* ^2^	0.43		0.05		0.44	
*F*	98.23***		6.91***		76.60***	

*p < 0.05, **p < 0.01, ***p < 0.001*p < 0.05, **p < 0.01, ***p < 0.001.

A mediation model was used to test the mediating role of mindful parenting. The results showed (see Table 4) that the 95% confidence interval for the mediating relationship between parenting stress and young children’s social–emotional competence via mindful parenting was [−0.033, −0.001], and the interval did not contain zero. Thus, mindful parenting plays a partial mediating role between parenting stress and young children’s social–emotional competence.

**Table 4:** Total effect, direct effect, and total indirect effect.

Effect	Effect value	*SE*	LLCI	ULCI
Total effect	−0.48	0.03	−0.537	−0.429
Direct effect	−0.47	0.03	−0.521	−0.412
Total indirect effect	−0.02	0.01	−0.033	−0.001

#### 3.3.2 Tests of the moderating effect of family socioeconomic status

Model 5 from the Hayes procedure was used to test the moderating role of family socioeconomic status. The results showed (see Table 5) that the interaction term of parenting stress and family socioeconomic status significantly and negatively predicted young children’s social–emotional competence ( *β* = −0.03, *p* < 0.005, 95% CI = [−0.050, −0.002]). Thus, family socioeconomic status played a significant moderating role in the association between parenting stress and children’s socioemotional strength.

**Table 5:** Mediation effect test results with moderation.

Variable	Equation: Young children’s social–emotional competence			
	*β*	*SE*	*t*	95%CI
Parenting stress	−0.47	0.03	−16.26***	[−0.528, −0.414]
Mindful parenting	0.72	0.03	2.51**	[0.156, 0.128]
Parenting stress x family socioeconomic status	−0.03	0.01	−2.17**	[−0.050, −0.002]
Family socioeconomic status	0.01	0.01	1.01	[−0.007, 0.021]
*R* ^2^	0.46			
*F*	56.04***			

*p < 0.05, **p < 0.01, ***p < 0.001.

**Figure 2:**
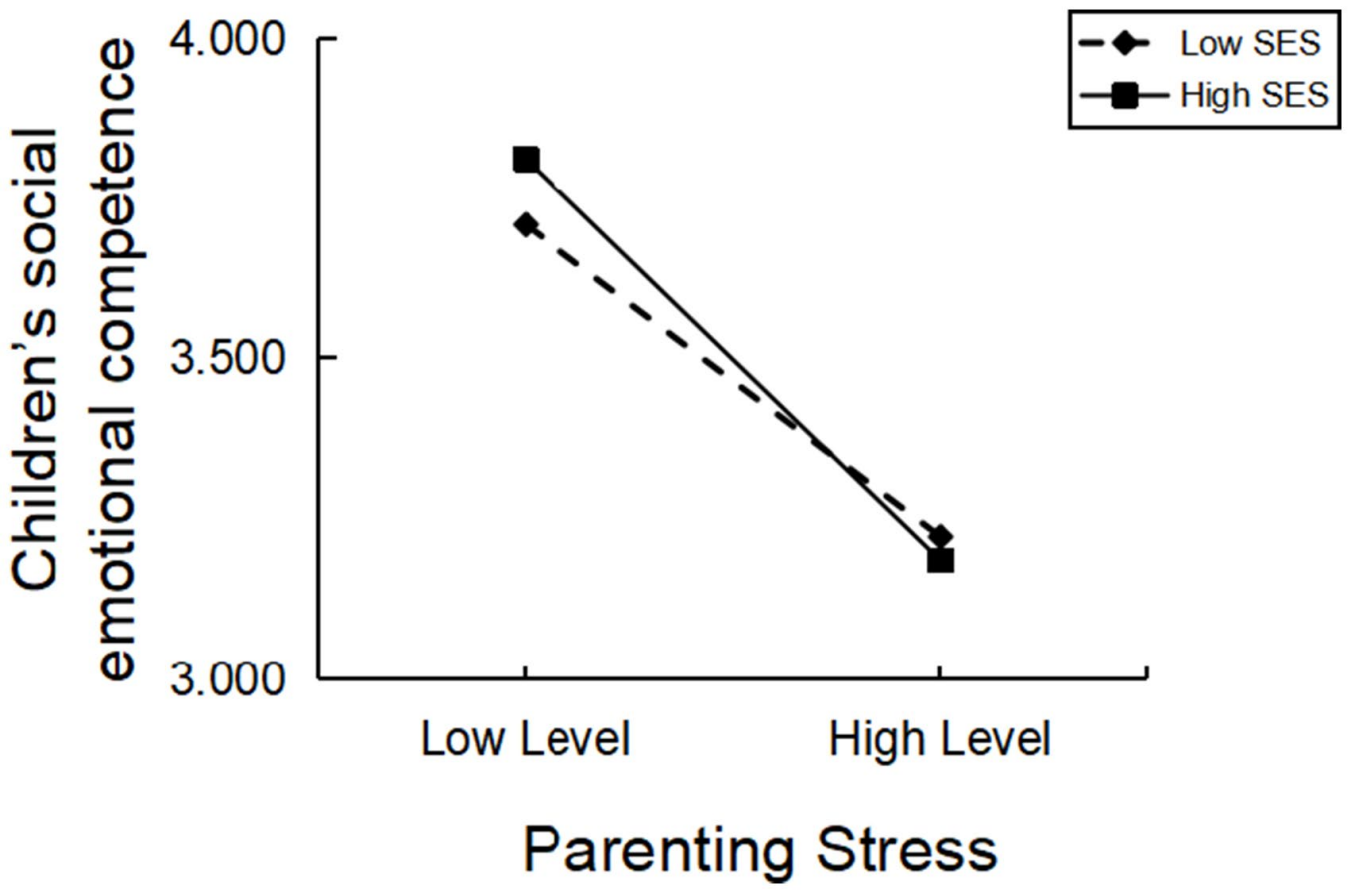
Moderating role of SES in parenting stress on young children’s social–emotional competence.

To better visualize the association between parenting stress and young children’s social–emotional competence at different levels of family socioeconomic status, the point-selecting method was used to estimate the predictive effect of parenting stress on young children’s social–emotional competence at high levels of family socioeconomic status ( *M + 1SD* ) and low levels of family socioeconomic status ( *M-1SD* ), respectively. The results, as shown in Figure 2, showed that parenting stress significantly and negatively predicted young children’s social–emotional competence in the group with high family socioeconomic status, and parenting stress similarly and significantly negatively predicted young children’s social–emotional competence in the group with low family socioeconomic status; in the high SES group ( *M + 1SD* ), parenting stress significantly negatively predicted young children’s social–emotional competence ( *simple slope* = −0.46 *SE* = 0.04 *t* = −12.7 *p* < 0.01), in the low SES group ( *M − 1SD* ), parenting stress also significantly negatively predicted young children’s social–emotional competence ( *simple slope* = −0.35 *SE* = 0.03 *t* = −10.99 *p* < 0.01).

## 4 Discussion

This study explores the potential association pathways linking parenting stress to young children’s social–emotional competence, examines the mediating role played by mindful parenting, and the moderating role played by family socioeconomic status. Based on the PSI-SF manual cut-offs (score ≥90th percentile), 112 families (27.9%) were classified into the high parenting stress group, while the remaining 290 families (72.1%) comprised the moderate stress group. The results showed that higher parenting stress was associated with lower levels of mindful parenting, which was associated with poorer development of social–emotional competence in young children. At the same time, family socioeconomic status plays an inverse moderating role in the effect of parenting stress on young children’s social–emotional competence.

### 4.1 The impact of parenting stress on young children’s social–emotional competence

The present study found that parenting stress was significantly and negatively associated with young children’s social–emotional competence, which is consistent with previous findings (Ortiz and Barnes, 2019). The family serves as the first environment in which children socialize and acquire emotional competence, and parents have a tremendous influence on the development of children’s social–emotional competence (Santelices et al., 2021). The potential pathway underlying this association is that when parents experience higher levels of parenting stress, they may exhibit changes in their emotional and behavioral patterns. Specifically, high parenting stress is often accompanied by negative parenting, which translates into a range of negative parenting styles, including low warmth and low positive emotional expression, over control, suspicion, and even extreme behaviors such as child abuse, such negative parenting styles create an emotional climate that elicits negative emotions and insecurity in children, which in turn is associated with poorer development of their social–emotional competence (Liu et al., 2015). Attachment theory suggests that the early parent–child relationship plays a crucial role in the development of children’s social–emotional competence. Patterns of interaction between parents and children, especially parents’ reactions and attitudes under stress, can profoundly affect children’s views of others and the world. When parents are unable to provide stable, positive parent–child interactions due to parenting pressures, children may develop insecure attachments. This insecure attachment pattern not only affects children’s understanding of and response to the emotions of others but also hinders the normal development of their social–emotional competence (Wang, 2024).

### 4.2 Mediation of mindful parenting

Mindful parenting mediates the association between parenting stress and young children’s social–emotional competence; that is, parenting stress is not only directly associated with children’s social–emotional competence but also linked to it through positive mindful parenting. On one hand, the finding that parenting stress negatively affects their level of mindful parenting is consistent with the results of existing research (Gouveia et al., 2016). The reason for this may be that difficult feelings such as stress generated by parents during the parenting process increase their expression of negative emotions and decrease their sensitivity, leading to lower levels of mindful parenting (Li et al., 2020). In addition, under high parenting stress, parents may be more inclined to ostracize and over-control children, show less enthusiasm, and develop negative patterns of interaction with children (Webster-Stratton, 1990). On the other hand, mindful parenting, as a positive parenting style, significantly contributes to the development of social–emotional competence in young children. Through mindful parenting, children can learn to use effective communication skills to find solutions, manage misbehavior, and regulate self-emotions in conflict situations (Ndengeyingoma et al., 2024). These competencies happen to be direct strategies for enhancing social–emotional competence, which is directly related to children’s understanding and expression of emotions, as well as their ability to interact socially (Wang and Yuan, 2024). Thus, mindful parenting not only provides a more harmonious and supportive environment for children but also lays a solid foundation for the overall development of their social–emotional competence.

### 4.3 Modulation of family socioeconomic status

The results revealed a nuanced moderating role of family socioeconomic status (SES). Specifically, SES moderated the direct effect of parenting stress on children’s competence, yet it exerted no moderating impact on the indirect pathway via mindful parenting. In other words, the mediating mechanism of mindful parenting remained consistent across SES strata, whereas the magnitude of parenting stress’s direct negative effect on children’s competence varied with family SES. Notably, although a moderating effect of SES was statistically detected, its coefficient was very small (*β* = −0.03). This indicates that in the sample of this study, while the moderating role of SES was statistically detectable, its actual influence was very limited. The negative impact of parenting stress on socioemotional abilities was widespread across families with different SES levels, but it was not effectively buffered by high SES. This finding may be related to the prevalent educational anxiety in China, where parents may experience intense parenting stress regardless of their family’s economic background. Notably, compared to low socioeconomic status groups, the negative impact of parenting stress on socioemotional development was more pronounced in high socioeconomic status groups. These findings contradict both the initial hypothesis and previous research results (Kao et al., 2018). Potential explanations include: first, parents from high socioeconomic backgrounds often impose stricter social expectations and more directive behaviors, which may hinder the development of children’s social skills. Second, children from affluent families are more susceptible to parenting stress, as evidenced by phenomena such as “Haidian moms,” “chicken parenting,” and “tiger moms” in Chinese online media. These practices reflect the inherent parenting stress in high socioeconomic status families, which is often transmitted to children through subtle mechanisms such as excessive intervention and emotional detachment in parent–child interactions.

Although this study delved into the effects and mechanisms of parenting stress on young children’s social–emotional competence, the following shortcomings still exist: first, this study took a cross-sectional approach, did not use a longitudinal study to dynamically observe and analyze the effects of parenting stress on the long-term development of social–emotional competence in young children. Second, a major limitation is the failure to test alternative causal paths. Cross-sectional data cannot rule out the reverse impact of children’s social–emotional competence on parental stress and mindful parenting. Third, the sample was only from Hebei Province, limiting the generalizability of results. Finally, this study relied heavily on the questionnaire method for data collection and analysis and did not use other research methods for deeper inquiry and causal inference.

## Data Availability

The raw data supporting the conclusions of this article will be made available by the authors, without undue reservation.

## References

[ref1] Abidin R. R. (1995). Parenting stress index: Professional manual. Odessa, FL: Psychological Assessment Resources.

[ref2] Anthony L. G. Anthony B. J. Glanville D. N. Naiman D. Q. Waanders C. Shaffer S. (2005). The relationships between parenting stress, parenting behaviour and preschoolers’ social competence and behaviour problems in the classroom. Infant and Child Dev. 9, 551–564. doi: 10.1017/s0954579497001302, 9327239

[ref3] Bradley R. H. Corwyn R. F. (2002). Socioeconomic status and child development. Annu. Rev. Psychol. 53, 371–399. doi: 10.1146/annurev.psych.53.100901.135233, 11752490

[ref4] Cassells R. C. Evans G. W. (2017). “Ethnic variation in poverty and parenting stress” in Parental stress and early child development: Adaptive and maladaptive outcomes, Cham: Springer International Publishing. 15–45.

[ref6] Chernyshenko O. Kankaraš M. Drasgow F. (2018). “Social and emotional skills for student success and well-being: conceptual framework for the OECD study on social and emotional skills”, OECD Education Working Papers, No. 173. Paris: OECD Publishing.

[ref7] Conger R. D. Conger K. J. Martin M. J. (2010). Socioeconomic status, family processes, and individual development. J. Marriage Fam. 72, 685–704. doi: 10.1111/j.1741-3737.2010.00725.x 20676350 PMC2910915

[ref8] Conger R. D. Donnellan M. B. (2007). An interactionist perspective on the socioeconomic context of human development. Annu. Rev. Psychol. 58, 175–199. doi: 10.1146/annurev.psych.58.110405.085530, 16903807

[ref9] Deater-Deckard K. (2008). Parenting stress. New Haven: Yale University Press.

[ref10] Duncan L. G. Coatsworth J. D. Greenberg M. T. (2009). A model of mindful parenting: implications for parent-child relationships and prevention research. Clin. Child. Fam. Psychol. Rev. 12, 255–270. doi: 10.1007/s10567-009-0046-3, 19412664 PMC2730447

[ref11] Gouveia M. J. Carona C. Canavarro M. C. Moreira H. (2016). Self-compassion and dispositional mindfulness are associated with parenting styles and parenting stress: the mediating role of mindful parenting. Mindfulness 7, 700–712. doi: 10.1007/s12671-016-0507-y

[ref12] Huang B. (2024).Influence of mindful parenting on adolescent interpersonal mindfulness: moderated mediation analysis.MA dissertation. Yangtze University. doi:10.26981/d.cnki.gjhsc.2024.001074.(In Chinese)

[ref13] Kabat-Zinn J. (1994). Wherever you go there you are: Mindfulness meditation in everyday life. New York: Hyperion.

[ref14] Kao K. Nayak S. Doan S. N. Tarullo A. R. (2018). Relations between parent EF and child EF: the role of socioeconomic status and parenting on executive functioning in early childhood. Transl. Issues Psychol. Sci. 4, 122–137. doi: 10.1037/tps0000139

[ref15] Kuo Y. L. Casillas A. Walton K. E. Way J. D. Moore J. L. (2020). The intersectionality of race/ethnicity and socioeconomic status on social and emotional skills. J. Res. Pers. 84:103905. doi: 10.1016/j.jrp.2019.103905

[ref16] Li X. Lam C. B. Chung K. K. H. Cheung R. Y. M. Leung C. Fung W. K. (2020). Development and validation of the Chinese inventory of children’s socioemotional competence (CICSEC). Early Educ. Dev. 31, 854–872. doi: 10.1080/10409289.2020.1715735

[ref17] Liang X. Wang Z. Yu J. (2021). Family socioeconomic status and toddlers’ social adjustment in rural-to-urban migration and urban families: the roles of maternal sensitivity and attachment security. Psychol. Dev. Educ. 37, 792–799. doi: 10.16187/j.cnki.issn1001-4918.2021.06.05

[ref18] Liu Y. Deng H. Zhang G. Liang Z. Lu Z. (2015). Association between parenting stress and child behavioral problems: association between parenting stress and child behavioral problems. Psychol. Dev. Educ. 31, 319–326. doi: 10.16187/j.cnki.issn1001-4918.2015.03.09 (In Chinese)

[ref19] Matthews K. A. Gallo L. C. (2011). Psychological perspectives on pathways linking socioeconomic status and physical health. Annu. Rev. Psychol. 62, 501–530. doi: 10.1146/annurev.psych.031809.130711, 20636127 PMC3121154

[ref20] McCabe P. C. Altamura M. (2011). Empirically valid strategies to improve social and emotional competence of preschool children. Psychol. Schs. 48, 513–540. doi: 10.1002/pits.20570

[ref21] Miyamoto K. Huerta M. C. Kubacka K. (2015). Fostering social and emotional skills for well-being and social progress. Eur. J. Educ. 50, 147–159. doi: 10.1111/ejed.12118

[ref22] Ndengeyingoma A. Jacob M. H. Beaulieu-Kratchanov V. Séguin M. (2024). Parenting competencies supporting the development of social and emotional skills of children—a scoping review. Trends Psychol 32, 425–448. doi: 10.1007/s43076-022-00194-3

[ref23] Ortiz R. M. R. Barnes J. (2019). Temperament, parental personality and parenting stress in relation to socio-emotional development at 51 months. Early Child Dev. Care 189, 1978–1991. doi: 10.1080/03004430.2018.1425297

[ref24] Ponnet K. Wouters E. Mortelmans D. Pasteels I. De Backer C. Van Leeuwen K. . (2013). The influence of mothers’ and fathers’ parenting stress and depressive symptoms on own and partner’s parent-child communication. Fam. Process 52, 312–324. doi: 10.1111/famp.12025 23763689

[ref25] Pearlin L. I. (1989). The sociological study of stress. J. Health Soc. Behav. 30, 241–256. doi: 10.2307/2136805 2674272

[ref26] Raikes H. A. Thompson R. A. (2005). Efficacy and social support as predictors of parenting stress among families in poverty. Infant Mental Health J. 26, 177–190. doi: 10.1002/imhj.20044, 28682501

[ref27] Ren C. (2010). Measurement methodology on social economic status index of students 5, 77–82. doi: 10.14082/j.cnki.1673-1298.2010.05.010

[ref28] Santelices M. P. Tagle F. Immel N. (2021). Depressive symptomatology and parenting stress: influence on the social-emotional development of pre-schoolers in Chile. Children 8:387. doi: 10.3390/children8050387, 34068229 PMC8153166

[ref29] Stone L. L. Mares S. H. W. Otten R. Engels R. C. Janssens J. M. (2016). The co-development of parenting stress and childhood internalizing and externalizing problems. J. Psychopathol. Behav. Assess. 38, 76–86. doi: 10.1007/s10862-015-9471-1 27069304 PMC4789299

[ref30] Tan T. X. Camras L. A. Deng H. Zhang M. Lu Z. (2012). Family stress, parenting styles, and behavioral adjustment in preschool-age adopted Chinese girls. Early Child Res. Q. 27, 128–136. doi: 10.1016/j.ecresq.2011.04.002

[ref31] Wang J. Yuan Y. (2024). Developing social and emotional competence of early child in New Zealand: guidance framework and project practice. Stud. Early Child. Educ. 3, 12–24. doi: 10.13861/j.cnki.sece.2024.03.001 (In Chinese)

[ref32] Wang L. Liang W. Zhang S. Jonsson L. Li M. Yu C. . (2019). Are infant/toddler developmental delays a problem across rural China? J. Comp. Econ. 47, 458–469. doi: 10.1016/j.jce.2019.02.003

[ref33] Wang X. (2024).Parenting-style and social and emotional competence in elementary school students: the mediating effect of parent-child attachment and moderating effect of peer relationships.MA dissertation. Shandong Normal university. doi:10.27280/d.cnki.gsdsu.2024.001107.(In Chinese)

[ref34] Wang Y. Xiao B. Li Y. (2023). Maternal mindfulness and preschool children’s social skills in China: the role of parenting stress and authoritative parenting behaviors. Curr. Psychol. 42, 30757–30766. doi: 10.1007/s12144-022-04084-w

[ref35] Webster-Stratton C. (1990). Stress: a potential disruptor of parent perceptions and family interactions. J. Clin. Child Psychol. 19, 302–312. doi: 10.1207/s15374424jccp1904_2

[ref36] Xiong H. Mao Y. Guan D. Tian J. (2023). The influence of principal leadership on students’ social-emotional competence——the chain mediating effect of teachers’ social-emotional beliefs and social-emotional competence. Educ. Res. Mon. 7, 60–68. doi: 10.16477/j.cnki.issn1674-2311.2023.07.003 (In Chinese)

[ref37] Xu Y. Zhang H. Liu Y. (2024). A moderated mediation effects of belief in a just world, family socioeconomic status and cognitive reappraisal on adolescent empathy. Chin. J. Clin. Psychol. 32, 1071–1074+1079. doi: 10.16128/j.cnki.1005-3611.2024.05.022 (In Chinese)

[ref39] Zhou H. Long L. (2004). Statistical remedies for common method biases. Adv. Psychol. Sci. 12:942.

